# Innovations in Health Care—A Conceptual Framework

**DOI:** 10.3390/ijerph181910026

**Published:** 2021-09-24

**Authors:** Steffen Flessa, Claudia Huebner

**Affiliations:** Department of General Business Administration and Health Care Management, University of Greifswald, 17489 Greifswald, Germany; claudia.huebner@uni-greifswald.de

**Keywords:** adoption, diffusion, digital health, e-health, implant, innovation, innovation management, personalized medicine

## Abstract

Innovations are the source of all human development and improvement of quality of life. At the same time, they challenge existing standards, solutions and societal patterns. In health care in particular, innovations enable us to treat previously incurable diseases or to make better use of scarce resources. However, they also make existing health care technologies obsolete, force specialists to learn completely new methods and require high investments. Consequently, in this paper we develop a conceptual framework model for the development, adoption and diffusion of innovations in health care. We analyse barriers and promoters of innovations, in particular meta-stability, costs, innovative ability and leadership and apply the framework to three innovations: personalized medicine, digital health, and implants. We conclude that strategic innovation management in healthcare is a prerequisite of the rapid development and adoption of innovations and the improvement of quality of life of the (aging) population.

## 1. Introduction

The terms “innovation” and “innovative” are buzzwords that are widely used in many different fields, including health care. To date, however, there is no comprehensive and generally accepted definition of innovation [1] and different sciences (e.g., economics, public health, geography, sociology) apply slightly different concepts. Schumpeter was one of the first economists to recognize the high relevance of innovations to every economic system ranging from a single business unit to entire economies, and to the world economy [2]. He described innovation as any change in the mode of production, manufacture of new products, company structures or entry into a new market and as the “creative destruction” that underlies all advances in a capitalist market regime [3].

Innovation is not the same as invention. While the invention describes the first emergence of a new idea or product, an innovation can be seen as the initial commercial implementation of a new idea as well as the economic optimization of knowledge utilization [4]. Accordingly, a narrower innovation term relates to successful market introduction [5]. In a market economy, invention and innovation can be distinguished, but not really separated, i.e., a free market ensures that an invention has a chance to prove its potential to become a new standard, but this does not necessarily mean that it will eventually prevail [6]. A distinction can be made between the invention as the generation of ideas and the first technical realization (e.g., a prototype) [7] and the innovation as the more comprehensive process starting with the generation of ideas and ending with the successful acceptance by potential users (adoption) [8], but both concepts are closely linked.

In the health care sector, innovations are the source of any improvement in the quality of services and quality of life, but also a steady challenge to existing health care providers and systems. Progress in medicine requires new technologies (e.g., drugs, implants and devices), procedures (e.g., new surgical techniques) or forms of organisation (e.g., palliative medicine as an innovative form of care). The tremendous increase of the quality of life and the length of life over the last 100 years can be attributed to innovations in health care or related fields, such as hygiene and nutrition. Innovation is constantly improving prevention. For instance, the new mRNA-vaccines allow us primary prevention of Covid-19, while the detection of circulating tumour cells [9] permits the implementation of secondary prevention. In addition, innovations revolutionize curative care. For example, stem cell transplants allow us to cure previously fatal cancers, and Zolgensma enables us to treat spinal muscular atrophy, which used to be a “death sentence”.

Despite the abundance and increasing need for innovations in health care, theoretical scientific research in this area is still very limited [10]. At the same time, the call for research on healthcare innovations is growing stronger. Although early conceptual papers can be found in the literature [11,12], many of them focus on specific health care applications, such as the pharmaceutical services [13]. Complementary innovations challenge existing solutions and systems and, therefore, find resistance within the established system. The main problem facing the health sector is not the scarcity of innovation, but the dissemination of innovative concepts [14]. The high costs of innovations, the necessity to learn new techniques and change existing systems as well as the fear of being replaced by new technology (e.g., radiological diagnostics will build-up barriers against innovation). Even if the invention seems fascinating, there is no guarantee that it will ever have success and become a new standard solution. In reality, many good ideas or products flop or in a small niche because they cannot overcome the barriers against them.

Consequently, there is a need to understand the innovation adoption process and to implement systematic innovation management in health care. This health care innovation management must cover the entire process from the first idea to a new standard of diagnostics or treatment. In this paper we develop a conceptual framework to understand the innovation process as well as the promotion and the barriers within this process. For this purpose, it is necessary to distinguish between different types of innovations and analyse their likelihood of adoption. In the next section, we will provide a typology of innovations. Afterwards, we will discuss the adoption and diffusion of innovations. These concepts are applied to three innovations (personalized medicine, digital health and implants). We close with conclusions on how innovation management in health care can be improved.

## 2. Typology of Innovations

In this section, we would like to differentiate innovations according to the object, the relationship to the existing standard, the system affected, the extent of change, and the readiness level. For each subtype, we will analyse whether an innovation is likely to make its way to become a new health care standard [4].

### 2.1. Object of Innovation

The first differentiation can be made according to the object of innovation, i.e., we ask the question “What is being innovated?” In principle, the term innovation is very, very broad and includes ideas, paradigms, scientific theorems, products, services, processes, organisational set-up etc. A product innovation implies that a new material product or tangible goods are invented and introduced into the market [15]. Service innovations are almost identical, but relate to intangible goods (e.g., new forms of logistics, advice etc.) [16]. Product and service innovations will only be successful if they satisfy needs of customers who were not at all satisfied with other goods or satisfied to a higher degree with the new product or service [1,16].

One problem with developing a product innovation is that frequently the time of development is rather long. New technical and medical knowledge challenges the original invention and thus makes it necessary to adapt the product even before the innovation is marketed. In this context, the concept of open innovation becomes increasingly relevant. This innovation approach is defined as the opening of the innovation process for system elements that are not directly involved in development and can thus significantly increase the potential of a novelty [17].

Process innovations change the way a product or service is produced [4]. The good remains (almost) unchanged, but the agents of production or the transformation of these agents into the existing products is altered. Process innovations frequently include a new production technology or a new business model. In a wider sense, a new process or structural organisation can be a process innovation as well. However, new products frequently require new technologies, i.e., product and process innovations can be distinguished, but not really separated.

Sometimes innovations go beyond certain products or technologies; they affect and involve the whole of society and can be described as social innovation [18]. For instance, adapting the health care system to the Primary Health Care Revolution expressed by the World Health Assembly in the Alma Ata Declaration in 1978 would be a social innovation [19].

A process innovation usually does not imply any major changes outside of the organisation. For instance, introducing a new software for operating room scheduling only affects the staff of a hospital, but neither patients nor anybody outside the hospital will realize that. Thus, resistance to process innovation is limited to one’s own staff. A new service (e.g., outpatient operations instead of inpatient services) affects the patients and their relatives. As a result, many more people are involved and can become obstacles in the adoption process. Social innovations often affect the entire population; therefore, they will find much more resistance to their implementation. The Primary Health Care Innovation finally failed not because of a bad process or product, but because of strong lobby groups that opposed in the shift of priorities within health services [20].

### 2.2. Relation to Existing Standard Solution

A product or social innovation can compete with an existing standard solution. Thus, potential customers will have to analyse whether the innovative alternative offers added value compared to the existing, known solution [21]. Every change comes at a cost. They can be of a financial nature (e.g., adopt the existing infrastructure to the new product) and of a non-financial nature (e.g., mental stress in the transition period from old to new). Consequently, a potential customer will only accept the competing innovation if it satisfies the needs to a higher extent than the old solution and if this benefit is worth the financial and non-financial costs. For instance, micro invasive operations could replace traditional surgery in many operations as the customers strongly appreciate the reduction in pain, length of stay and costs. Some micro invasive operations (e.g., cataract surgery) have become a new standard, shifting traditional surgery to small niches (e.g., for the very old).

Other innovations bring something completely new to this world, i.e., the new product or service satisfies a need that was previously completely uncovered [21]. For instance, stem cell implantations can heal cancers that were inevitably fatal before. There is very little resistance to these enriching innovations unless their cost is very high. Enriching innovations usually also require process innovations, in particular new production technologies.

### 2.3. System Level Affected

**Figure 1:**
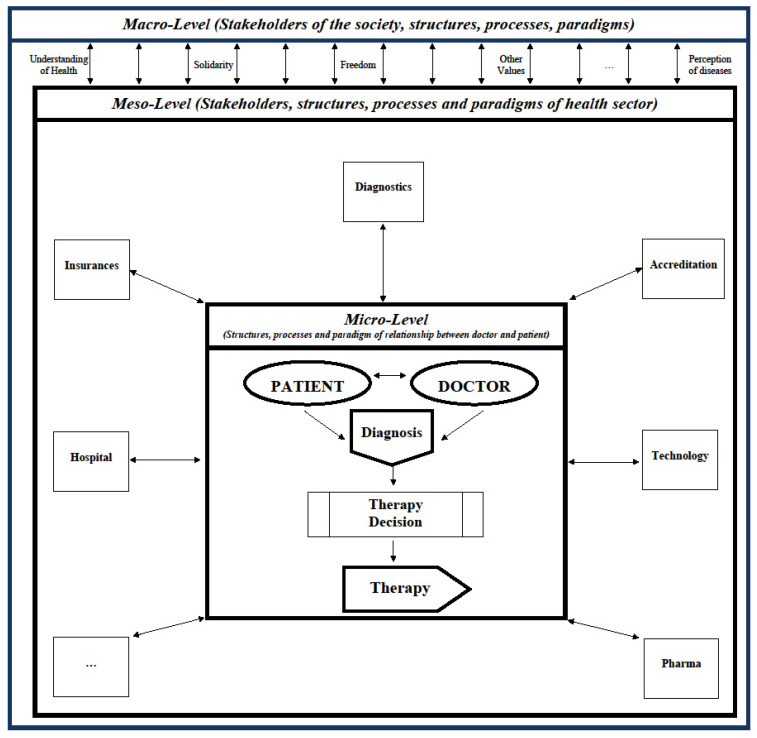
Micro-, Meso- and Macro-Innovation.

An innovation can only affect a small sub-system of the base, the total system or even the supra-system. Accordingly, a distinction can be made between micro-, meso- and macro-innovations [22]. Figure 1 exhibits the three system levels, which can be affected by a diagnostic or treatment innovation. At the micro-level, only the structures, processes and paradigms of the doctor-patient relationship are affected [23]. For instance, an alternative and innovative hip replacement has no effects on the hospital management, the health insurance company or the patient’s relatives, unless the costs are higher and / or are not covered by the health insurance company. Here, however, not only the expenditure for the innovation but the total costs must be considered, i.e., also the follow-up costs (e.g., due to the need for rehabilitation) and possibly the opportunity costs (e.g., due to longer operation times per replacement).

The meso-level comprises all stakeholders, structures, processes and paradigms of the health care sector [22,23]. This frequently includes insurance companies, diagnostic and technology enterprises, accreditation boards, and the pharmaceutical industry. For instance, if the hip replacement is also drug-releasing, it will most likely be much more expensive and not covered by the existing DRG (diagnosis related group) rebate. As a result, not only the physician and his patient are involved in introducing this innovation, but also the hospital management, insurance company and most likely other legal entities who need to design a new DRG group specifically for drug-eluting hip implants.

On the macro-level, all stakeholders, structures, processes and paradigms of the society are involved [22,23]. In particular, social values constitute the framework in which the health care system (meso-level) and the doctor-patient relationship (micro-level) persist. Values such as freedom, equity, and solidarity, as well as fundamental paradigms such as our understanding and perception of health and disease are quite constant. However, the COVID-19 pandemic has shown that the disruptions in the health system are sometimes so severe that they affect society as a whole with its value system and basic paradigm. A health problem—mostly regulated in the health system—suddenly affected civil rights and changed our understanding of solidarity between age groups.

It is obvious that micro-innovations are usually more easily adopted, while macro-innovations have a very difficult time becoming a new standard. Therefore, most innovations are on the micro-level, but from time to time the perturbance gets so severe that the micro-level cannot absorb it with smaller innovations involving only very few sub-systems. Then the meso-level has to take care of it, e.g., the management of a hospital. This frequently involves other elements of the health care system. It is only rarely that these system elements cannot accommodate the changes so that pressure is exerted on the macro structure. If an innovation requires a change in the macrostructure, it requires extremely strong pressure or it takes a very, very long time.

In addition, it should be emphasized that the three levels do not act independently of one another, but are very closely linked and thus influence one another. An example of this would be the influence of a pharmaceutical company (agent at the meso-level) on a doctor’s perception of an innovative drug in the context of sponsored clinical studies. This, in turn, could affect the relationship between doctor and patient at the micro-level. Similar influences would be exerted through health policy lobbying on the macro-level. This could distort innovation processes, i.e., accelerate or even slow them down.

### 2.4. Extent of Change

In business administration (including the management of health care providers such as hospitals), the extent of change is of great importance in assessing innovations [24]. Radical innovations involve significant changes. If a hospital decides to build a nursing home, this is a radical innovation for that hospital. A very radical innovation that opens up a completely new market is sometimes called a disruptive innovation. For instance, the new Covid-19 mRNA-vaccines are disruptive innovations with the potential to become not only a new market, but also the new standard for all other vaccines.

However, the majority of innovations are incremental ones. They optimize existing products or technologies in order to adapt them to the (changing) needs of the customer and/or reduce production costs. Sometimes new laws require incremental innovation. It is obvious that the resistance against incremental innovations is rather low in comparison to disruptive innovations shaping a completely new market. Frequently, incremental innovations are made within a business unit, while disruptive inventions are often made in start-ups. Existing enterprises buy these start-ups in order to acquire the innovation, make them ready for the market and finally become a champion of this product [25]. For instance, so-called companion drugs (drugs that are only sold together with genetic tests) have become a new standard of treatment in some fields of cancer treatment, but the pharmaceutical companies had no experience in molecular diagnostics. Consequently, they bought start-ups in order to add the innovation to their portfolio [26].

### 2.5. Readiness

**Figure 2:**
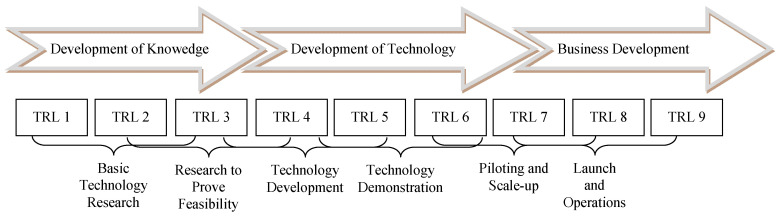
Technology Readiness Level, own illustration based on [28].

Finally, the proximity to the market or the technology readiness helps to distinguish innovations. Some innovations are still in a very early stage of their development, while others are currently being launched on the market and are fighting for the new standard. The Technology Readiness Level (TRL) [27] was first designed for the development of aerospace technology and is often used in industrial innovation processes. As shown in Figure 2, it assesses the level of development on a scale from 1 (very early basic research) to 9 (established system with validation and proof of success). While a TRL of 1 to 3 mainly serves to generate knowledge, a product on level 4 to 6 expresses technology development. From level 7, the product is close to the market and subject of business development.

A major problem of innovation management in the health care sector is that physicians, engineers and natural scientists are completely responsible for basic research and partly also for technology development, while health care managers are in charge of the business development. There is little cooperation and oversight over the entire process with little integration. Systematic innovation management is a rare exception. Consequently, some products are developed and consume huge investments, although early market analysis could have indicated that their market chances are limited. Obviously, the TRL increases the likelihood of becoming the standard. However, any organisation needs a profound portfolio of innovations at different readiness levels in order to always have a sufficient number of future “stars” to survive on the market. This is well understood in the pharmaceutical and medical technology industry, but hardly implemented in the service production field. For instance, there is hardly any structured and fully synchronized innovation management in hospitals [29].

Based on these differentiations, we can state that some innovations are very likely to be adopted, in particular enriching micro-innovations that satisfy a human need at low costs and without forcing other systems to adjust as well. Macro-innovation and innovations, which have to compete with existing and successful standard solutions, will face much more resistance, even so strong that they might not diffuse at all. Consequently, it is worthwhile to analyse the process of adoption and diffusion of innovations in detail.

## 3. Adoption and Diffusion of Innovations

**Figure 3:**
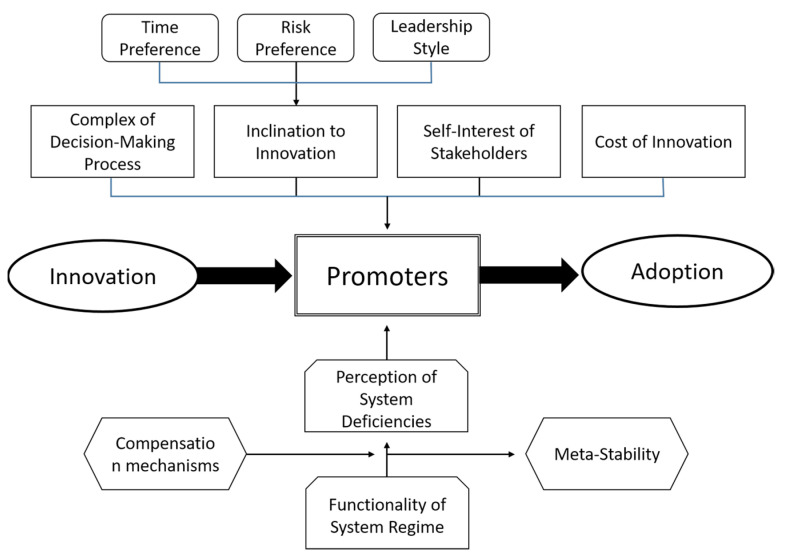
Model of Adoption of a Health Care Innovation.

Figure 3 exhibits a model of the adoption of a health care innovation. It assumes that a new idea, paradigm, product, service, or technology was invented elsewhere and has evolved to the point where an organisation or any other system could adopt and implement it. The model answers the question of what has to happen in order for this innovation from outside to become an accepted innovation from inside the organization. The so-called promoters are in the center of the analysis. They are the key persons who finally make the adoption happen [30]. Different types of promoters have different roles. With their expertise, expert promoters contribute to overcoming the barrier of lacking know-how [31]. They know “how things work”, they understand the innovation and see its perspectives [32]. However, even a competent expert promoter is insufficient to lead the adoption process to success. The power promoter helps to overcome the barrier of unwillingness. He makes the decision that the innovation is introduced into the enterprise and that sufficient resources are allocated to the adaption and development process [1]. He has the power to break resistance within the organisation.

Hauschildt and Chakrabarti indicate that even expert and power promoters might not suffice for a successful adoption process, as administration might be another obstacle which cannot be overcome [31,33]. For instance, legal obligations might kick out the power and expert promoters, i.e., even a great innovation might be rejected if the data protection officer vetoes it. Thus, a successful adoption process needs an administrative promoter. In addition, some authors call for a relationship promoter constantly looking for innovation seedlings from outside that might be relevant for the organisation [31,34] in order to overcome barriers of lacking or misguided communication between the organization, customers and other stakeholders [35].

The model indicates that these promoters are only willing to support an innovation if they perceive system deficiencies. If everybody is satisfied with the current solution, there is no need to risk an innovation. Only if the existing system solution is dysfunctional will promoters dare to incur the costs of an alternative. However, the normal reaction to a dysfunctional system is not to change it completely, but to repair it. Thus, potential promoters will first attempt to improve the existing solution instead of adopting a completely new alternative. They search for compensation mechanisms, such as improving the design, reducing the factor costs, or simply reducing their own expectations. In order to avoid risking a complete system change, managers tend to uphold the existing solution for years with strong financial and mental support. The consequence is an artificially stabilized solution, which can only exist as long as the managers are willing to shield it. However, the stability of the system is artificial, it is meta-stabile [21]. Unless managers shield the old solution from the pressures of dysfunction, it will collapse. Consequently, the adoption process is not linear (the more dysfunctional an old system is, the faster the adoption rate). For quite a long time, nothing might happen. Then suddenly the organisation accepts an innovation like a jump to a higher level.

In addition, four factors influence the willingness and ability of the manager to adopt an innovation. Firstly, the complexity of the new solution determines the likelihood and speed of the adoption. Highly complex innovations, which require a lot of explanation and involve many elements and relations, are unlikely to be adopted. This is one reason why macro-innovations are less easily adopted than micro-innovations. It is the role of the expert promoter to reduce the complexity and the resulting uncertainty [36].

Secondly, the costs of the innovation are crucial for the likelihood and speed of adopting it. As already mentioned in Chapter 2.3, the term cost does not only cover financial expenditure to develop and implement the new technology. Instead, it covers the non-financial costs of reduced productivity during the transformation of the old to the new system. It also includes the negative consequences of the limited intellectual performance of managers who concentrate on the new methodology. Another issue are the political costs in the sense of resistance against the innovation (even co-workers going on strike against it) [37]. Micro-innovations that solve a problem that everyone considers crucial and without existing system solutions will take hold much more easily, especially if they do not require large investments.

Thirdly, the inclination of promoters and other stakeholders towards innovation is a crucial factor as well. It depends on three factors, i.e., time preference, risk preference and the leadership style within the organization. The time preference is an expression of the value of future. If a person wants to see results today and disrespects future benefits, he has a high time preference and will not adopt an innovation because most innovations show their competitive advantage against the standard solution only after some time [38]. The risk preference expresses whether a decision-maker likes, accepts, or hates risks. If a person is very much afraid of taking a risk, he will always stay with the current solution. Even if it is bad, it might still be better for him than taking the risk that the innovative alternative might fail. Finally, adopting an innovation always requires that employees have the mental space to test and experiment, try and fail, try again and succeed. A leadership style based on command and control that is completely based on obedience and strict task fulfilment therefore leaves no room for innovation. In their analysis of patient-centered innovation in health care organizations, Hernandez et al. also emphasize the importance of internal primary determinants, including effective leadership [11]. Consequently, a person has a high probability of becoming a promoter if they have a low time preference and a low risk appetite and, in addition, work in an organisation with a coaching and fostering leadership style. [1,4,37].

Fourthly, stakeholders only become promoters if the innovation does not contradict their own self-interest [39]. For instance, the famous Alma Ata Declaration was completely against the self-interest of the majority of the most prominent healthcare workers, medical doctors. While everybody agreed that Primary Health Care is an ideal solution, in particular for resource-poor countries, it was obvious that medical doctors would lose their unique position if the focus shifts from big hospitals to preventive work outside health care such as building latrines and planting of vegetables for proper nutrition [20].

The adoption of an innovation into an organisation and into a complete system is a highly complex process in which many stakeholders and influence factors are involved [11]. Often enough, excellent ideas, technologies or products do not find sponsors because the dysfunctionality of the existing system is not yet sufficient, the deficiencies within the existing solution can be partially remedied, the costs of the conversion are viewed as too high because the change is being made harm a powerful influence group, or there are no executives who can or want to accept the innovation [14,29]. In these cases, the innovation might only be adopted in a small niche. Here it can mature and get rid of “teething troubles” so that it becomes a potential [40] or innovation seedling [21]. As soon as the old standard solution becomes more and more obsolete, the seedling is ready for complete takeover, and under the increasing pressure on the dysfunctional old solution, the new solution could become the new standard [41].

Innovation adoption does not only occur in a single organisation but also in an entire network, a branch, an economy or a region. However, not every organisation will adopt the innovation at the same speed. Due to the individual inclination to innovation (see above), different organisations might be innovators, early adopters, early majorities, late majorities or laggards [38]. However, innovations do also diffuse in space. Contagious diffusion means that an innovation spreads through a population by contact from one person to a neighbouring person, i.e., it is like a wave sweeping through a region. Relocation diffusion occurs when an innovation moves between two points attracting each other even over a longer distance [42]. Relocation diffusion often occurs in a hierarchy of organisations or locations (e.g., cities) where bigger institutions have a higher gravity and attract the phenomenon earlier than smaller places (hierarchical diffusion) [21]. The diffusion of an innovation in time and space is hierarchical and contagious at the same time leading to complex patterns of diffusion.

**Figure 4:**
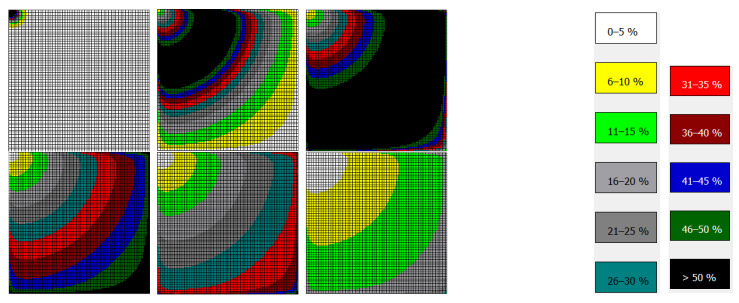
Contagious Diffusion in a Plain Space, for t = 34, 52, 65, 73, 78, 87.

Figure 4 shows an example of a very special “innovation” (COVID-19) moving as a wave through a plain space. It becomes obvious that the wave produces a characteristic pattern that resembles the pattern of other phenomena, such as fashion, smoking habits or water births. They take their origin in one place and spread through the entire region. At the most remote place, they may no longer be relevant at the beginning, but there may be another innovation. In healthcare, innovation cycles are longer than and not as drastic as in fashion, for instance, but they do exist.

In the following, we would like to explain the different types of innovations and the adoption model using three examples from the health care field.

## 4. Case Studies

### 4.1. Personalized Medicine

Although the term “Personalized Medicine” (PM) has been used for almost two decades, there is still no generally accepted definition. Most authors or institutions focus on the application of omics technologies (genomics, proteomics, and metabolomics) to diagnose, treat, and prognosticate a disease. For instance, the Personalized Medicine Coalition defines PM as “the use of new methods of molecular analysis to better manage a patient’s disease or predisposition to disease” [43]. Other authors include diverse biomarkers for medical decision-making [44]. PM tries to avoid the “one-size-fits-all” medicine, in which a particular diagnostic procedure, therapy or a prevention program is used for all patients. Instead, it wants to adjust these interventions specifically for one patient or a smaller group of patients based on the respective constellation of biomarkers.

PM is an innovation, but the readiness levels of diverse instruments of PM differ greatly. Genetic testing to find the most appropriate pharma intervention has become obligatory in some fields of oncology (pharmacogenomics [45,46]). In other medical areas, however, patient stratification is still in its infancy, where standard treatment regimens are usually based on a single-cause-single-effect model [47], in which one intervention solves the one single problem for all patients suffering from it [48]. Generally, PM is an early innovation and it is undecided whether it (like many other innovations) will disappear within a short period, persist in a narrow niche or become a new standard solution for medical decision-making across the health care sector.

Assessing the potential of PM requires that we judge whether it is a micro-, meso- or macro-innovation. A micro-innovation changes only the physician-patient relationship or direct prevention, diagnosis or treatment, i.e., no other stakeholders are affected and no structures of the health care system have to be adjusted to the innovation [23]. PM systems medicine has developed in certain niches (e.g., of oncology), where mainly physicians and molecular biologists are affected by their potential. The patients hardly notice the new technology, the hospital administration is almost unchanged, the financing system remains as it is and the public is not informed. Thus, PM is a micro-innovation, which makes it much easier to adopt in a small circle of experts. Whether it requires structural changes at the macro-level, whether it turns into a macro-innovation, depends on its ability to overcome barriers.

Furthermore, an enriching innovation is much more likely to be adopted than a competing innovation. PM can be seen as an enriching innovation as it opens up possibilities of diagnosis, treatment and prevention of diseases where no functional standard solution exists. The better the standard solution is functional, the lower the probability that an innovation will find promoters. However, we are aware that many chronic-degenerative diseases today can neither be cured with a single-cause-single-effect paradigm, nor can patients be restored to health with a one-size-fits-all medicine. PM promises to fight diseases that cannot be cured to this day. Thus, it is likely that PM will find promoters and become a standard in its current small fields of application. However, as time goes by it will diffuse further and challenge some of the existing standards, regulations, and paradigms of medicine. Outside of the rare disease oncology niche, and in the routine treatment of widespread chronic degenerative diseases, it will face resistance, as it will compete with standard medicine.

An innovation becomes the new standard solution if the existing solution is dysfunctional or the innovation is much better than the existing one [41]. In many cases, an innovation will not win the race against the standard solution. It will persist in a niche in which it matures into a potential [40] or an innovation seedling [22], whose adoption as a basic paradigm of health care is being oppressed. For a certain time the existing system solution will be stabilized artificially (meta-stability) [22] until the pressure becomes too strong to make the old system regime functional.

**Figure 5:**
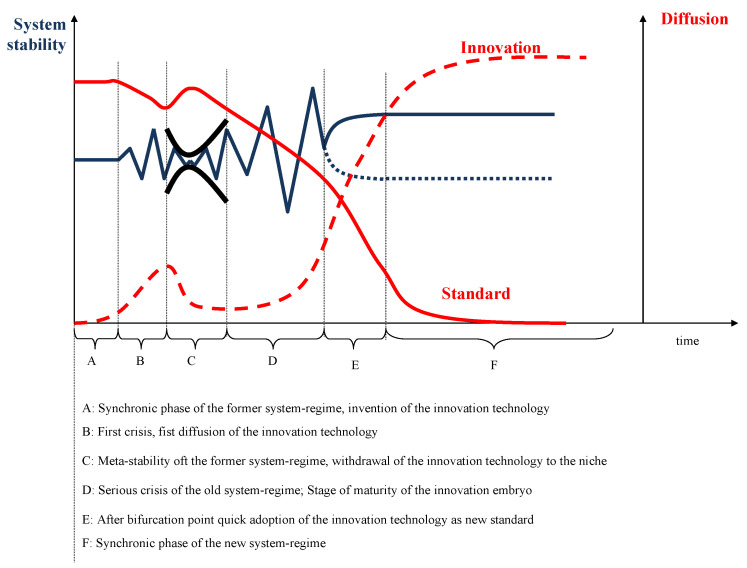
Change of the system regimes, following on [22].

Figure 5 illustrates the phases of the development from the old system regime to the new, innovative regime. In phase A (synchronic phase), the old system-regime (here: one-size-fits-all medicine) is stable. The innovation technology (here: PM) is invented. In phase B, the old regime faces constraints and comes into a first crisis. In this time, the innovation technology can start to diffuse as many see the shortcomings of the old system solution and look for alternatives. It is clear, however, that PM has far-reaching consequences, costs, and risks. Thus, most decision-makers attempt to improve the existing system (phase C) and stabilize the old regime. The system is artificially stabilized; it becomes meta-stabile. In this phase, the innovation technology withdraws to a niche, such as PM in oncology. When the population realizes that more and more diseases remain incurable with the traditional approach to medicine, the old regime comes into a severe crisis (phase D). Meanwhile, the innovation can mature and become an innovation seedling ready to take-over leadership. If the crisis is sufficiently severe, many decision-makers will adopt the innovation and reduce the older standard to niches (phase E). Finally, the innovation has become the new standard.

Figure 5 indicates that the adoption process is hardly ever a straightforward one. Instead, evolutionary jumps occur when the innovation technology has matured within a niche and rapidly becomes the new standard. For many who were not aware of the innovation seedling waiting in the niche, this new standard is a surprise. At the point of bifurcation, the existence of these potentials determines the direction of further development.

Whether PM actually becomes the new standard of health care or whether it remains a phenomenon in a small niche of very complex (oncological) diseases depends on the assessment of whether PM is a micro-, meso- or macro-innovation. Currently, PM is a micro-innovation, which does not really challenge the existing medical system. The vast majority of physicians and procedures in the health care field are not affected at all. Thus, it develops in the niches of scientific research and for some rare diseases. However, PM has the potential to become even more. One could envisage a time when medical decision-making is routinely based on a much broader dataset of individuals and epidemiological standards than it is today. There will be many obstacles in the way from remaining in a small niche to a new standard, i.e., the broad application would require new investments, new financing regulations, and a new self-image of physicians as decisions-makers in close collaboration with bio-statisticians and computer scientists.

However, PM has the potential to become a macro-innovation challenging the entire value system of society. The persisting paradigm of health and disease is that a person is sick when he has certain symptoms of a disease. This approach is binary, i.e., a person is sick or healthy and non-genetic risk factors alone are insufficient to allocate a person to the status “sick”. PM challenges this assumption, as it can lead to a different understanding of health and disease. A genetic test (e.g., for personalized prevention) implies the existence of a status “not-yet-sick”, i.e., a person has a genetic predisposition to a disease that may be coming but not developed. The occurrence of symptoms is associated with a certain degree of uncertainty, i.e., PM has a predictive value [49] which can challenge our binary understanding of heath and disease [50]. We do not deny that certain non-genetic risk factors can also be used to predict health outcomes, such as high cholesterol. However, the “not-yet-sick” status has not yet been established in society. A smoker continues to smoke even if the likelihood of developing lung cancer is high. Only when the diagnosis has actually been made or the first previous illnesses appear do people rethink and may stop smoking.

The respective redefinition could call into question further assumptions of our society. For instance, anyone with a high predictive value who opposes prevention can be considered “guilty” of his disease. Consequently, solidarity with the sick might deteriorate. The freedom of the individual (including the freedom to invest in prevention or not!) might end when the consequences of prevention are predictable on an individual level. Furthermore, some authors even argue that our anthropology might change. The technical possibility of developing unique therapeutic items will sooner or later raise the question of whether humans are more than just a warehouse of human spare parts—a challenge of our ethics [51].

### 4.2. Digital Health

Digital health can be defined as the deployment of information and communication technologies in the health care sector in order to improve prevention, diagnosis, treatment and management of processes and organisations that affect people’s health [52]. The most important component is E-health (electronic health), which applies information and communication technologies in the core of the health care system, in particular through web-based applications. Digital health is broader than E-Health as it also includes digital technologies in sports, fitness and wellness as long as they have a positive effect on health [53]. There is ample evidence that E-health, including tele-medicine, digital patient records, remote surgery etc. have the potential to significantly improve the health of the population, particularly in rural areas where specialized services are otherwise unavailable [54,55,56].

E-health is a rather young innovation. Although first attempts at tele-medicine were undertaken in the last century, its broad application required the availability of broadband internet to transfer (e.g., for imaging) or store data (e.g., cloud-based patient files) [57]. The technology and concepts can be imported into any country, so that one would expect that E-health had diffused to all countries, at least within the European Union, in a similar way. However, this is not a matter of fact.

In 2018, the Bertelsmann Foundation [58] published a digital-health index that includes different dimensions of digital health, i.e., infrastructure (e.g., nationwide patient identification number, technical data infrastructure), legal framework (e.g., data protection regulation, data security), institutionalisation (e.g., national digital health organisation, standards, financing) and specific applications (e.g., digital patient records, e-prescription, video consultations, health information, health research). Using the data of 17 OECD-countries, they calculated the Digital-Health-Index (DHI) shown in Table 1.

It is obvious that the adoption of the Digital Health innovation is very different in these 17 countries. This requires an analysis of the reasons. For this analysis, we will concentrate on EU countries and ignore Canada, Australia, Switzerland, and Israel. As Table 2 shows, there is hardly any correlation between country statistics and the DHI. For instance, one would expect that richer countries (expressed in terms of gross national income per capita) would have more resources to invest in digital health and thus, would be faster to adopt the innovation. However, this is not the case. It seems that the wealth of a country has hardly any impact on the acceptance of the innovation.

Quite surprising, population density also has no influence on the DHI. We could expect that countries with a lower population density would see the benefits of digital health (and in particular telemedicine) rapidly. Instead, there is a small correlation between DHI and the population as a whole, with digital health seeming to be more evident in countries with smaller populations, suggesting that smaller countries (in population) are more innovative or adaptable.

We did not expect digital health to have an impact on life expectancy, as this indicator reacts to structural changes with considerable delay. It might take many years before we realize that countries with a good digital health care system have better health outcomes. It is a surprising, however, that neither the health expenditure nor the share of the government and/or compulsory insurance in total health spending are correlated with DHI, i.e., other parameters must determine whether a country adopts the innovation rapidly.

The innovation adoption model exhibited in Figure 3 can provide some reasons for different patterns of adoption of digital health innovation. Firstly, the pressures on stakeholders may vary widely. Germany, for instance, has a well-functioning health care and social protection system, which has been in a steady-state systems regime for decades. Dysfunctionalities of the systems are usually “healed” within the system, i.e., rules and regulations are adjusted, and additional financial resources are provided without challenging major pillars of the system. Estonia, to the contrary, had to build-up a completely new health care and social protection system after its independence. There was no meta-stability of the old (mainly paper-based) system and Estonia could not afford it—at least for the first 20 years. Thus, Estonia started almost from scratch and could jump directly into a digital system [61,62].

Secondly, the propensity to innovate differs between countries, particularly in the areas of time preference, leadership style and risk aversion. Hofstede analysed the dimensions of national cultures [63,64]. In this initial work he distinguished power distance, individualism, avoidance of insecurity and masculinity; later he added long-term orientation and indulgence versus restraints [27]. As Table 3 shows, there is a negative correlation between power distance and DHI, i.e., cultures with a strict and hierarchical leadership style have a lower penetration of the health care system with digital technologies. Likewise, cultures in which dominance, assertiveness or win-lose-thinking are seen as virtues (“masculine cultures”) also tend to have a low DHI. The more people try to avoid uncertainty (and risks), the lower is the adoption of digital health. Other dimensions show less or almost no difference. It is obvious that the champions of the adoption of the digital health innovation all show a smaller power distance, a smaller masculinity and a smaller avoidance of uncertainty than the respective average, which underlines that the model shown in Figure 3 points to the correct influencing factors for the rapid adoption of innovations. Still, adequate statistical analysis would be needed to confirm the extent to which any of these factors contribute to digital health innovation. Therefore, further future research in this area will be funded.

In addition, there seems to be another institutional factor that strongly influences the willingness and ability to adopt the innovation. There are large differences in the individual willingness to share personal data in exchange for benefits or rewards, such as better health care services or lower insurance premiums [28]. While in some countries 40% of people are strictly against the disclosure of personal data (e.g., Germany), in other countries only 19% see this as a problem (Italy). Unfortunately, some countries of the sample given above were not included in the GfK-sample, but it indicates that data confidentiality levels differ strongly between countries. Scandinavian and Baltic countries seem less afraid of exchanging data. This is demonstrated, for instance, by the age of consent, i.e., the age from which children do not need the consent of their parents to give data on the internet. While it is 13 in Estonia, Sweden and Denmark (i.e., in countries with a high DHI), it is 16 in Poland, Germany and Netherlands.

Sliwa, Brem and Agarwal analysed the institutional contexts of E-health in Germany, Austria, and Denmark. They concluded that complexity and documentation requirements are major barriers to the implementation of telecare (Austria, Germany), while practical governmental regulations will support patient-centred and innovative solutions (Denmark) [57]. The role of data protection laws and willingness of individuals to share data in digital records has to be further analysed, but it is obvious that a brilliant idea (such as digital health care) cannot be adopted and diffused if cultural and legal barriers prohibit it. The model presented in Figure 3 can provide a framework for analysing diffusion patterns of innovations in health care.

### 4.3. Implants

Implants are medical devices that replace, support or enhance missing or damaged biological structures. Typical examples are hip prosthesis, pacemakers or cochlear implants. They are artificial devices and can be described as product innovations with high risks (becoming part of the human body) and costs (e.g., neurostimulator > 20,000 US$). As the life expectancy increases and the age of implantation (e.g., of cochlear implants) decreases, several implant changes are necessary in life due to the longer application time. This greatly increases the risk and lifetime cost of implants. Consequently, innovative implants attempt to be durable, avoid increasing loss of functionality and improve maintenance. Worldwide there is a wide market for innovative implants and the respective innovation process requires scientific management, but disruptive innovations are rare. Instead, most innovations are incremental, so the innovation has to compete with the existing implant (which was an innovation before!).

**Figure 6:**
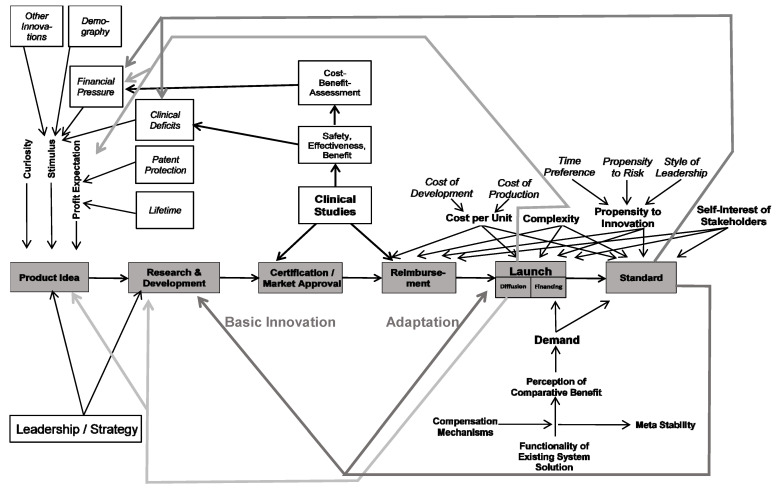
Phase Model of the Development of an Implant.

Figure 6 combines Figure 2 and Figure 3 and specifies these for the development of an implant from the first idea to a marketable solution. It indicates that the development process is never a linear path, but rather consists of an adaption cycle with multiple feedback loops [62]. It starts with the generation of a product idea and ends with the establishment of the innovative implant as part of the standard therapy solution. Implants go through several development phases before they are finally launched on the market. During development, new knowledge in the fields of medicine, biochemistry or engineering can be discovered that might require an early adaptation of the implant [63]. The information for this early adaption requires strong networks with outsiders, i.e., open innovation becomes highly relevant [12].

As discussed in Section 2.5, different phases can be distinguished. After a new implant idea has been generated, a prototype is developed and technical certification is achieved. These are more or less technical aspects, but they also require input from the market as well. Afterwards, most implants require that a national health service or insurance company accept the implant as part of their funded service package (reimbursement). Once this has been achieved, the market is much broader than for the (few) self-payers. The market launch can start after technical certification and reimbursement have been fixed, but even then, the product can still flop if there is no demand. Finally, the development of the innovative implant as a new standard must be analysed and controlled.

Certification and reimbursement require a little more attention. Most countries or groups of countries have certification bodies, such as the CE-marking in the European Union. They regularly require clinical trials to prove that the new device “has been assessed by the manufacturer and deemed to meet EU safety, health and environmental protection requirements” [65], i.e., the manufacturer has to prove that the new implant is effective, tolerant and safe by a risk-benefit-analysis [66]. Clinical trials are costly and time-consuming and can delay the market approval significantly. However, the risk of a dysfunctional implant for the patient is considerable and requires high standards of safety (medical device regulation). In Germany, for instance, medical devices are attributed to certain risk categories, with implants generally falling into the highest class (§ 137h SGB V, class III) and requiring a clinical trial [67].

Once the technical safety has been approved, the reimbursement must be secured. Especially in countries where the Social Health Insurance or a National Health Service covers the majority of the population (e.g., Germany and Great Britain), only reimbursable implant innovations have a chance of surviving on the market in the long term. Introduction to the market is fairly simple if the novel product is less expensive but has a similar benefit compared to the existing standard in therapy, which is already reimbursed [68]. In many cases, however, the innovative implant will be more expensive than the existing standard. In other cases, the innovative implant offers therapy for a previously incurable disease. In different countries, there are different rules for calculating a fair price for the innovative implant, but in most cases, this requires a long negotiation and often a cost-benefit analysis.

## 5. Conclusions

These few examples indicate that the development, adoption and diffusion of an innovation in health care cannot be taken for granted. The vast majority of innovations—great ideas with very good intentions—will never find their way into the health care market, even if they have the potential to meet people’s needs. Profit certainly plays a role, but to use the right tools and to have the knowledge of innovations are even more important. The process from the original idea to the establishment of the innovation as the new standard solution requires professional management, i.e., proper planning, thorough organisation, appropriate staffing, constant motivation and many forms of control and synchronisation.

Innovations in health care can be small (such as a new wrapping for sterilizing) or tremendous (such as a completely new paradigm of medicine and a shift in our understanding of health and disease). The existing world is the result of innovation processes of the past—and the future world will be the consequence of today’s innovation processes. Whether this future brings a better quality of life, more efficient health care provision and equity among stakeholders depends a lot on the inventions we make today, the adoption process, and current diffusion. Our innovations help determine whether future generations will still face the same medical problems that we do. This prospect should encourage us to invest more into proper innovation management that encompasses the entire spectrum from generating creative ideas to marketing the new products to the new standard. The starting point is a thorough analysis of the type of innovation, the phase in the innovation process, potential adopters and promoters of all kinds, barriers of implementation and a temporal and spatial diffusion path. The conceptual framework presented in this paper is designed to support this analysis.

## Figures and Tables

**Table 1:** Digital-Health-Index. Source: Own compilation based on data of Bertelsmann Foundation [58].

Rank	Country	DHI
1	Estonia	81.92
2	Canada	74.73
3	Denmark	72.47
4	Israel	72.45
5	Spain	71.36
6	England	69.98
7	Sweden	68.26
8	Portugal	67.19
9	Netherlands	66.05
10	Austria	59.81
11	Australia	57.31
12	Italy	55.81
13	Belgium	54.67
14	Switzerland	40.62
15	France	31.61
16	Germany	30.02
17	Poland	28.52

**Table 2:** Country Statistics (Selected OECD-countries). Source: Own compilation based on data of OECD and World Bank [59,60].

Country	GNI p.c. PPP (Current International $)	Population, Total	Population Density (p. km^2^)	Life Expectancy at Birth (yrs.)	Current Health Expenditure p.c. PPP (Current International $)	Government/Compulsory Health Spending in % of Total
Austria	58,940.00	8,917,205	107.1	81.79	5879.10	75.2%
Belgium	55,370.00	11,555,997	377.4	81.75	5404.92	76.0%
Denmark	62,180.00	5,831,404	144.8	81.20	5794.26	83.8%
Estonia	37,940.00	1,331,057	30.4	78.50	2427.63	74.4%
England	47,620.00	67,215,293	274.7	81.20	4619.57	77.8%
France	50,400.00	67,391,582	122.5	82.58	5250.45	83.7%
Germany	55,220.00	83,240,525	237.3	80.94	6098.20	85.0%
Italy	42,270.00	59,554,023	202.9	83.20	3624.08	74.1%
Netherlands	59,700.00	17,441,139	511.8	82.01	5634.53	82.7%
Poland	33,220.00	37,950,802	124.0	77.86	2015.29	70.3%
Portugal	33,980.00	10,305,564	112.3	80.70	3242.40	61.2%
Spain	42,250.00	47,351,567	93.7	83.49	3576.49	70.8%
Sweden	56,270.00	10,353,442	25.0	82.96	5828.41	85.2%
Correlation DHI	0.051	−0.595	−0.099	0.140	−0.069	−0.166

**Table 3:** Dimensions on Selection National Cultures. Source: own compilation based on GfK-data [28].

Country	Power Distance	Individualism	Masculinity	Uncertainty Avoidance	Long-Term Orientation	Indulgence
Austria	38	90	61	51	21	71
Belgium	65	75	54	94	882	52
Denmark	18	74	16	23	35	70
Estonia	40	60	30	80	82	16
England	35	89	66	35	51	69
France	68	71	43	86	63	48
Germany	35	67	66	65	83	40
Italy	50	76	70	75	61	30
Netherlands	38	80	14	53	67	68
Poland	68	60	64	93	38	29
Portugal	63	27	31	99	28	33
Spain	57	51	42	86	48	44
Sweden	31	71	5	29	53	78
Average	47	69	43	67	116	50
Correlation DHI	−0.47	−0.04	−0.53	−0.36	−0.07	0.22

## Data Availability

Data sharing not applicable.
